# LEARNING FROM LIFE STORIES: RECRUITING NURSING HOME RESIDENTS FOR A LIFE STORY WORK PROGRAM

**DOI:** 10.1093/geroni/igz038.1122

**Published:** 2019-11-08

**Authors:** Brian K Polk, Farida Ejaz, Miriam Rose

**Affiliations:** Benjamin Rose Institute on Aging, Cleveland, Ohio, United States

## Abstract

Recruiting nursing home (NH) residents to participate in program evaluations is a consistent challenge. This was evident in a federally supported project to improve person-centered care of long-stay NH residents enrolled in Medicaid. Evaluators sought to examine the impact of a life story work intervention using a pre-post study design involving interviews of NH residents and surveys of their family members and staff. Other resident eligibility criteria included willingness to participate in both research and life story interviews, age 60+, a Brief Inventory Mental Status (BIMS) score of 8 or higher, English-speaking, and consent from a legal guardian, if applicable. A total of 16 NHs agreed to participate in the implementation and evaluation of the program, which developed complimentary, individualized life story booklets for residents and a companion summary for staff. Of the homes’ combined population of 1,817 residents, 569 met eligibility criteria for the research study. Non-response from legal guardians excluded 37 residents, and 174 residents approached for recruitment declined to have their names released to the researchers. During baseline interviews, 20 residents failed the BIMS, 21 were unavailable, and 79 refused when approached by a research interviewer. Ultimately, 238 resident interviews were completed at baseline. Common themes for refusals included disinterest in participating in life story work, statements that theirs was not a good life worth talking about, and doubts that quality of care would improve. Strategies for addressing such challenges included displaying sample life story materials during recruitment and providing residents additional time to consider participation.

